# Supplementary figures and images for: DnaJC7 specifically regulates tau seeding
Source: eLife. 2023 Jun 30;12:e86936. doi: 10.7554/eLife.86936 (PMC10473839; doi:10.7554/eLife.86936)

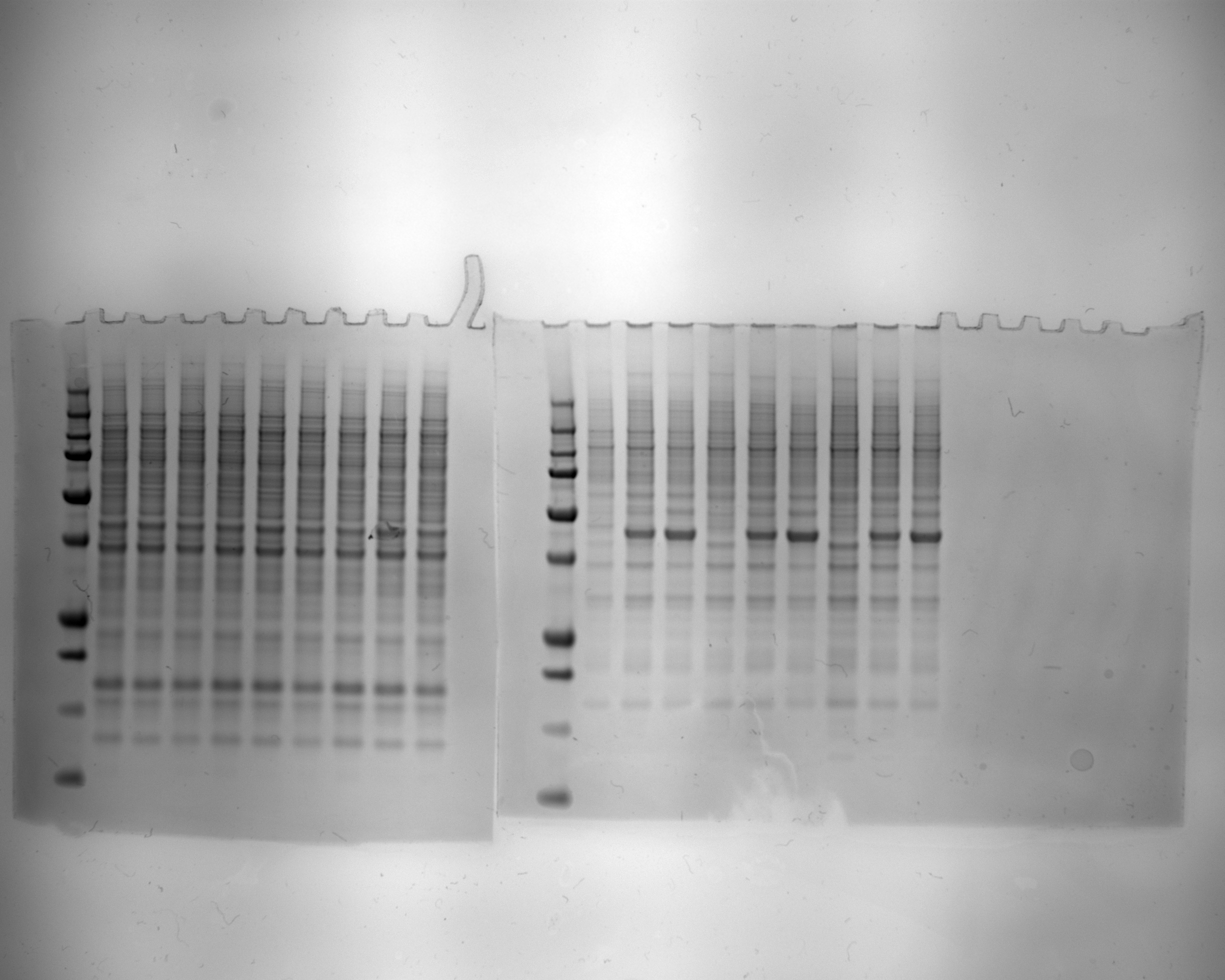

Supplement: Figure 1—figure supplement 1—source data 1. [file elife-86936-fig1-figsupp1-data1.zip › Figure 1 - Supplement 1 - Source Data 1/Figure 1 - Supplement 1 - Uncropped Original.tif]

## Supernatant Fraction

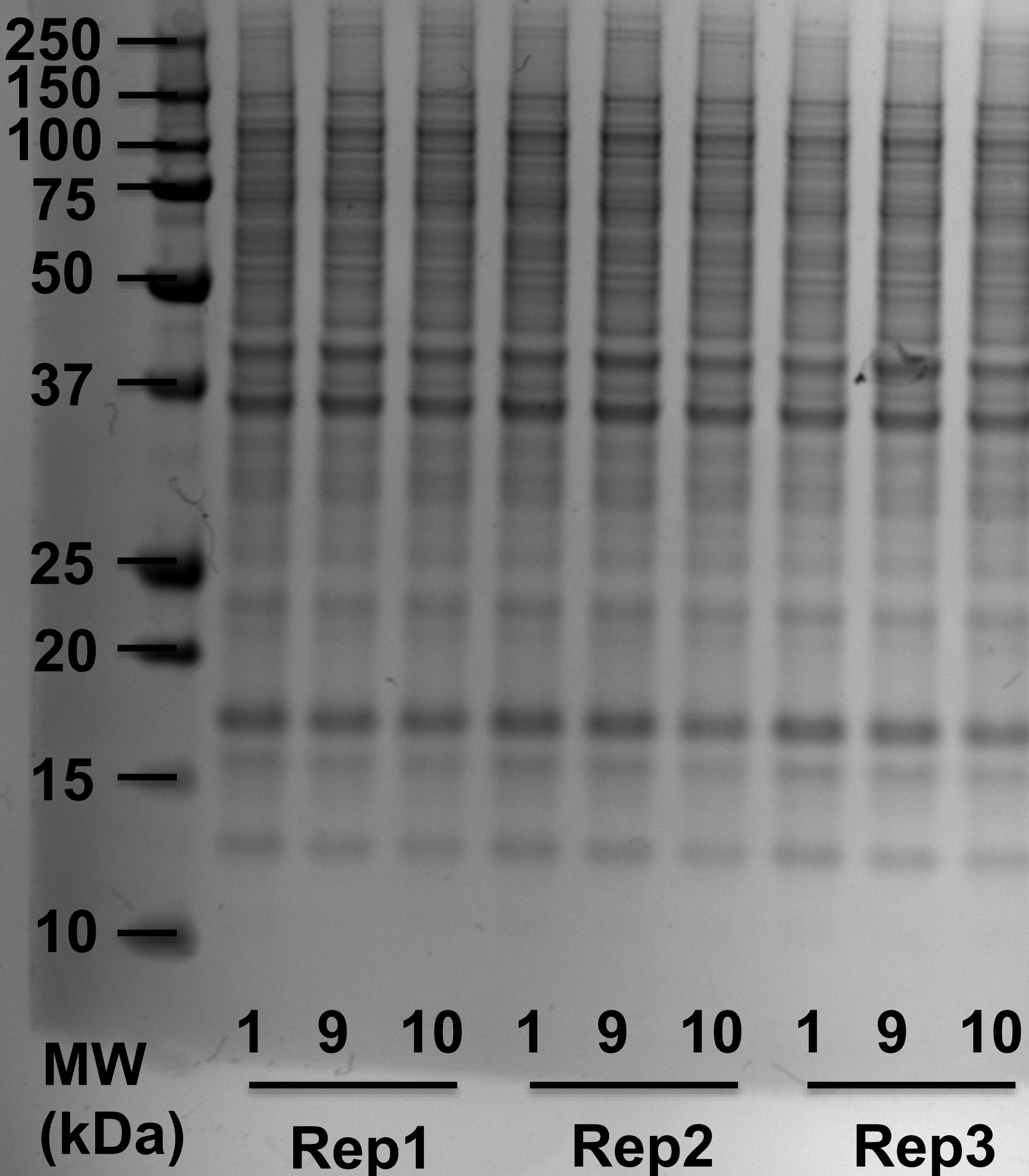

## Pellet Fraction

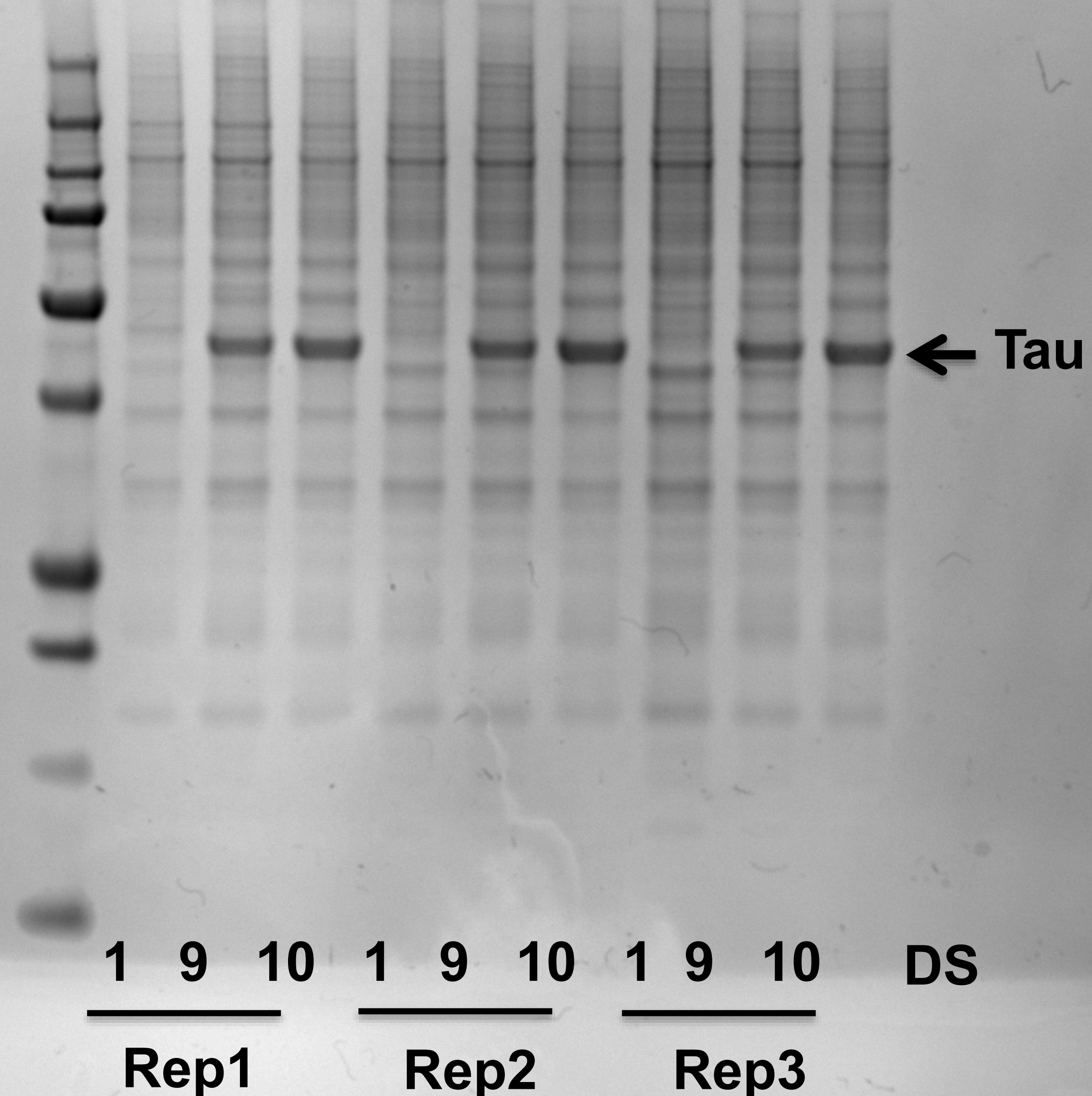

Mass Spec Analysis (Whole Lane)

Supplement: Figure 1—figure supplement 1—source data 1. [file elife-86936-fig1-figsupp1-data1.zip › Figure 1 - Supplement 1 - Source Data 1/Figure 1 - Supplement 1 - Uncropped Labelled.pdf]

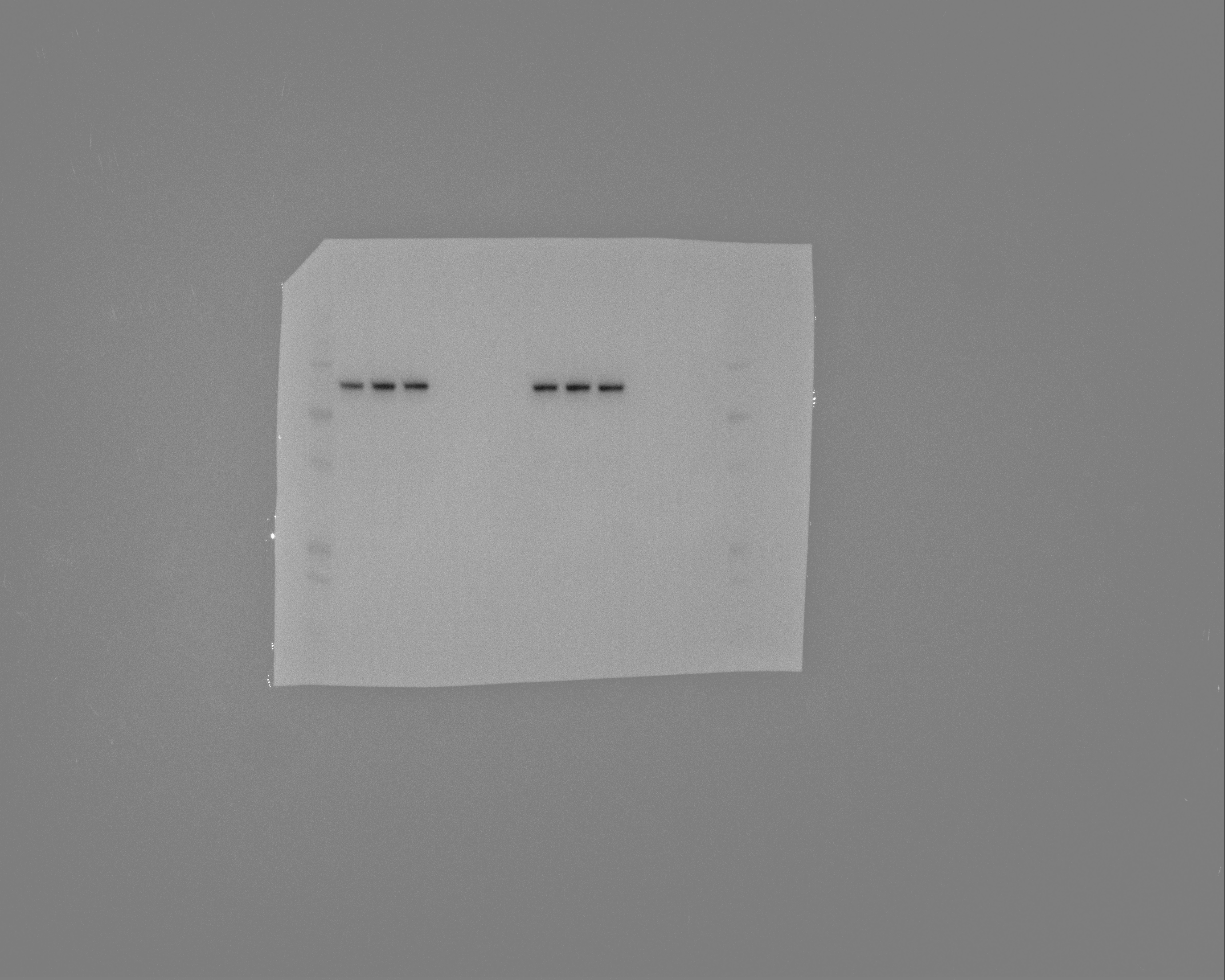

Supplement: Figure 2—figure supplement 1—source data 1. [file elife-86936-fig2-figsupp1-data1.zip › Figure 2 - Supplement 1 - Source Data 1/Figure 2 - Supplement 1 - Uncropped Original.tif]

$\alpha$ -DnaJC7

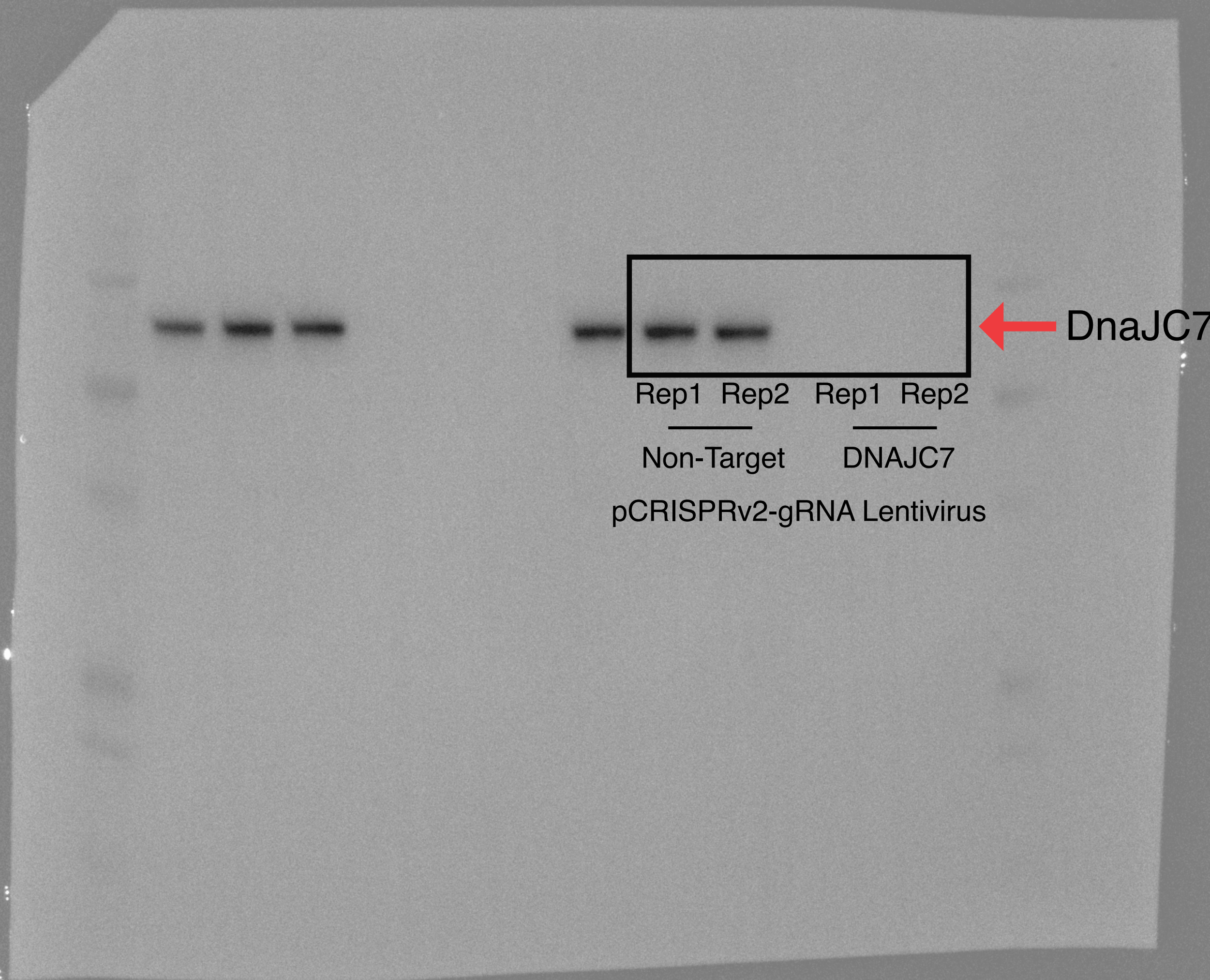

Rep1 Rep2 Rep1 Rep2

Non-Target DNAJC7

pCRISPRv2-gRNA Lentivirus

← DnaJC7

Supplement: Figure 2—figure supplement 1—source data 1. [file elife-86936-fig2-figsupp1-data1.zip › Figure 2 - Supplement 1 - Source Data 1/Figure 2 - Supplement 1 - Uncropped Labelled.pdf]

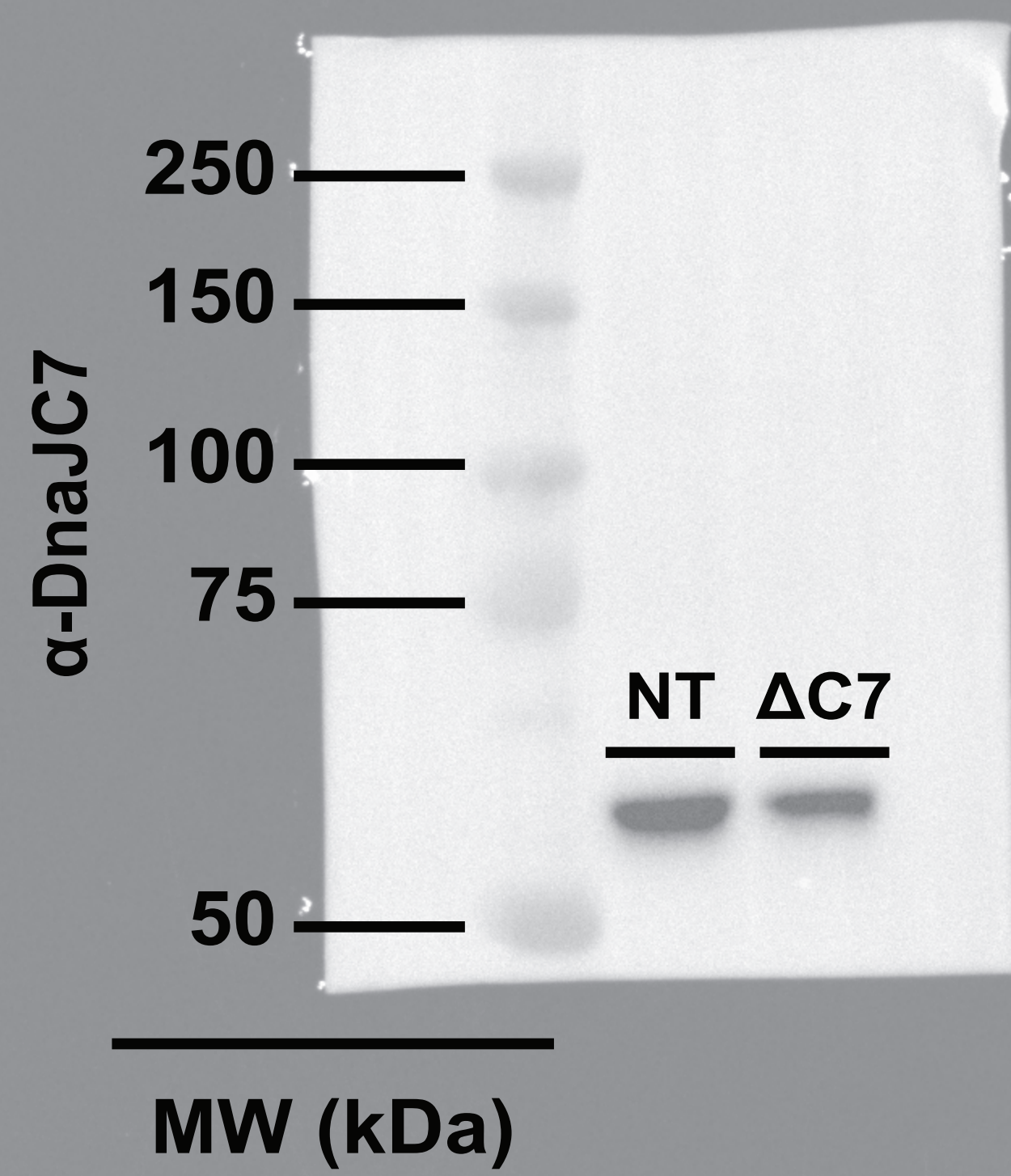

Supplement: Figure 3—figure supplement 1—source data 1. [file elife-86936-fig3-figsupp1-data1.zip › Figure 3 - Supplement 1 - Source Data 1/Uncropped Labelled/Panel C - DnaJC7 blot.pdf]

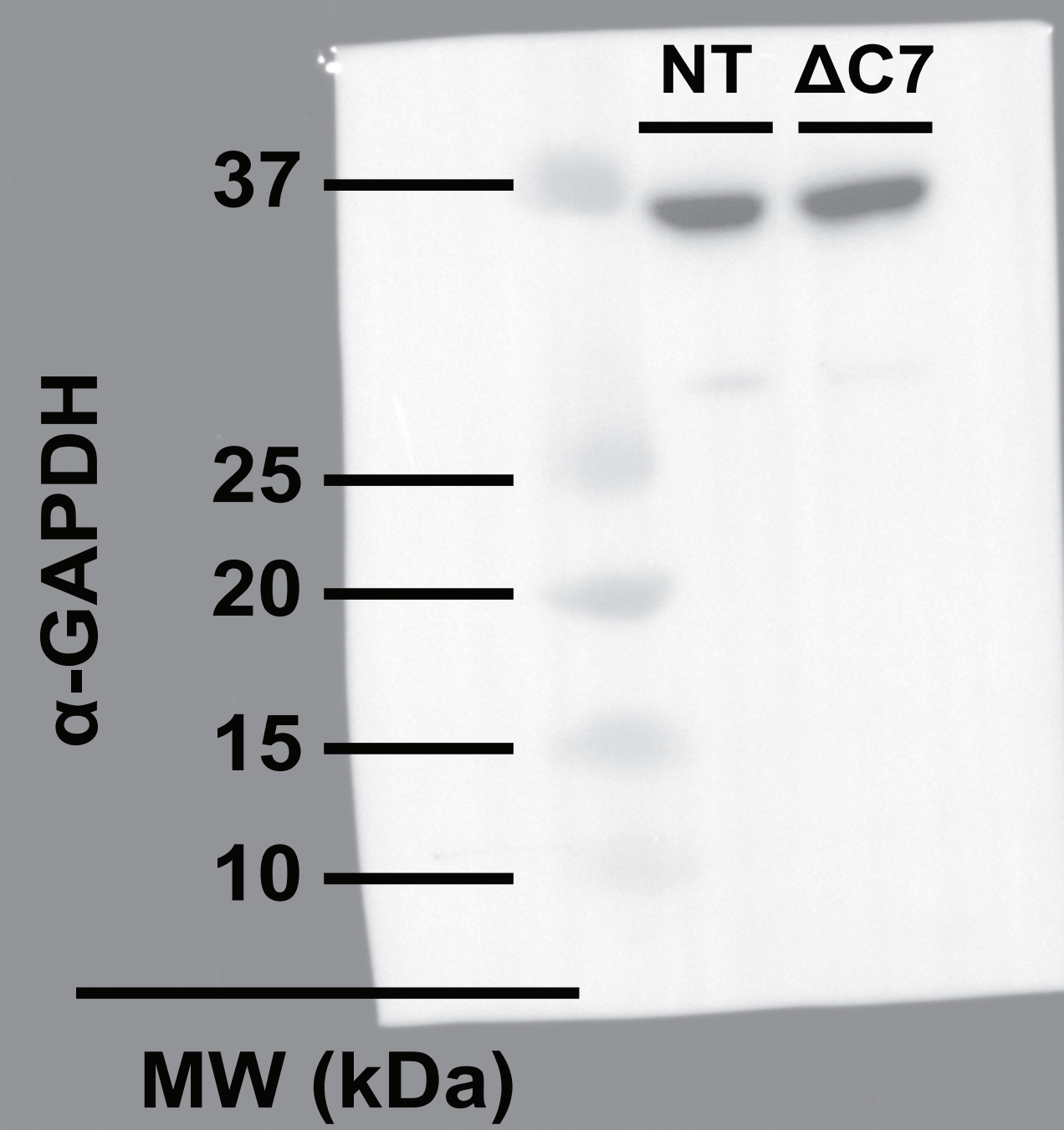

Supplement: Figure 3—figure supplement 1—source data 1. [file elife-86936-fig3-figsupp1-data1.zip › Figure 3 - Supplement 1 - Source Data 1/Uncropped Labelled/Panel C - GAPDH blot.pdf]

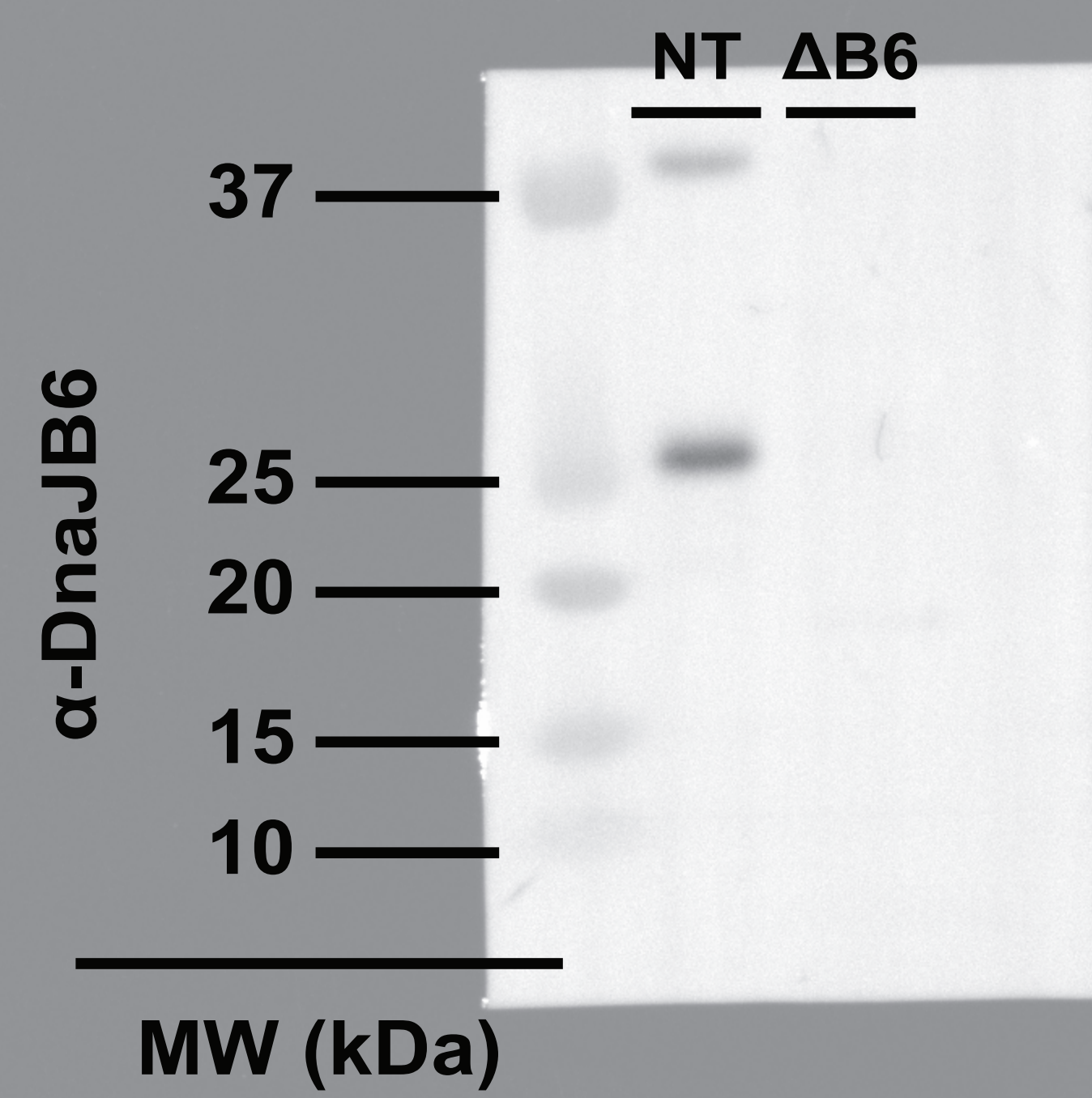

Supplement: Figure 3—figure supplement 1—source data 1. [file elife-86936-fig3-figsupp1-data1.zip › Figure 3 - Supplement 1 - Source Data 1/Uncropped Labelled/Panel C - DnaJB6 blot.pdf]

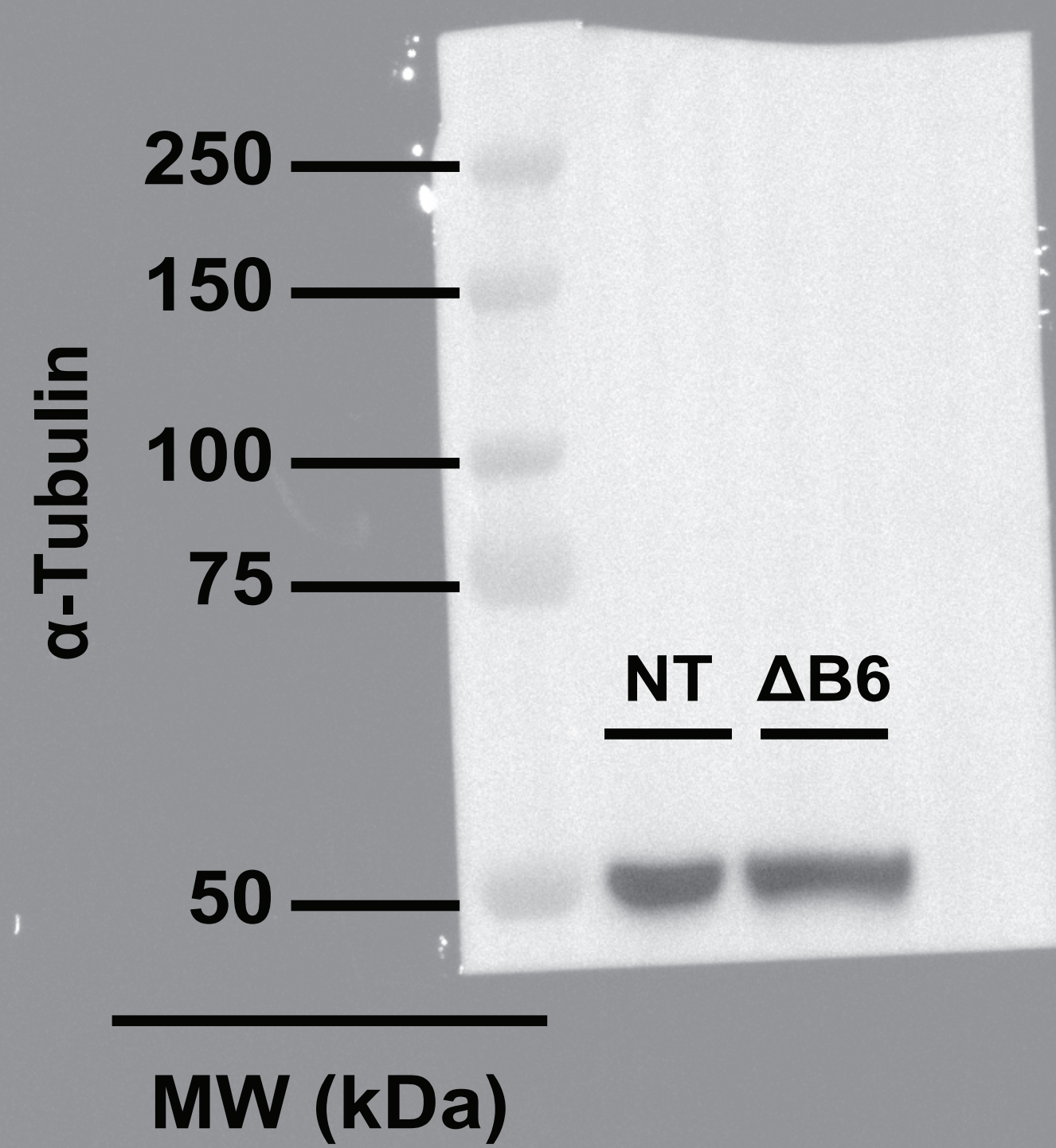

Supplement: Figure 3—figure supplement 1—source data 1. [file elife-86936-fig3-figsupp1-data1.zip › Figure 3 - Supplement 1 - Source Data 1/Uncropped Labelled/Panel C - Tubulin blot.pdf]

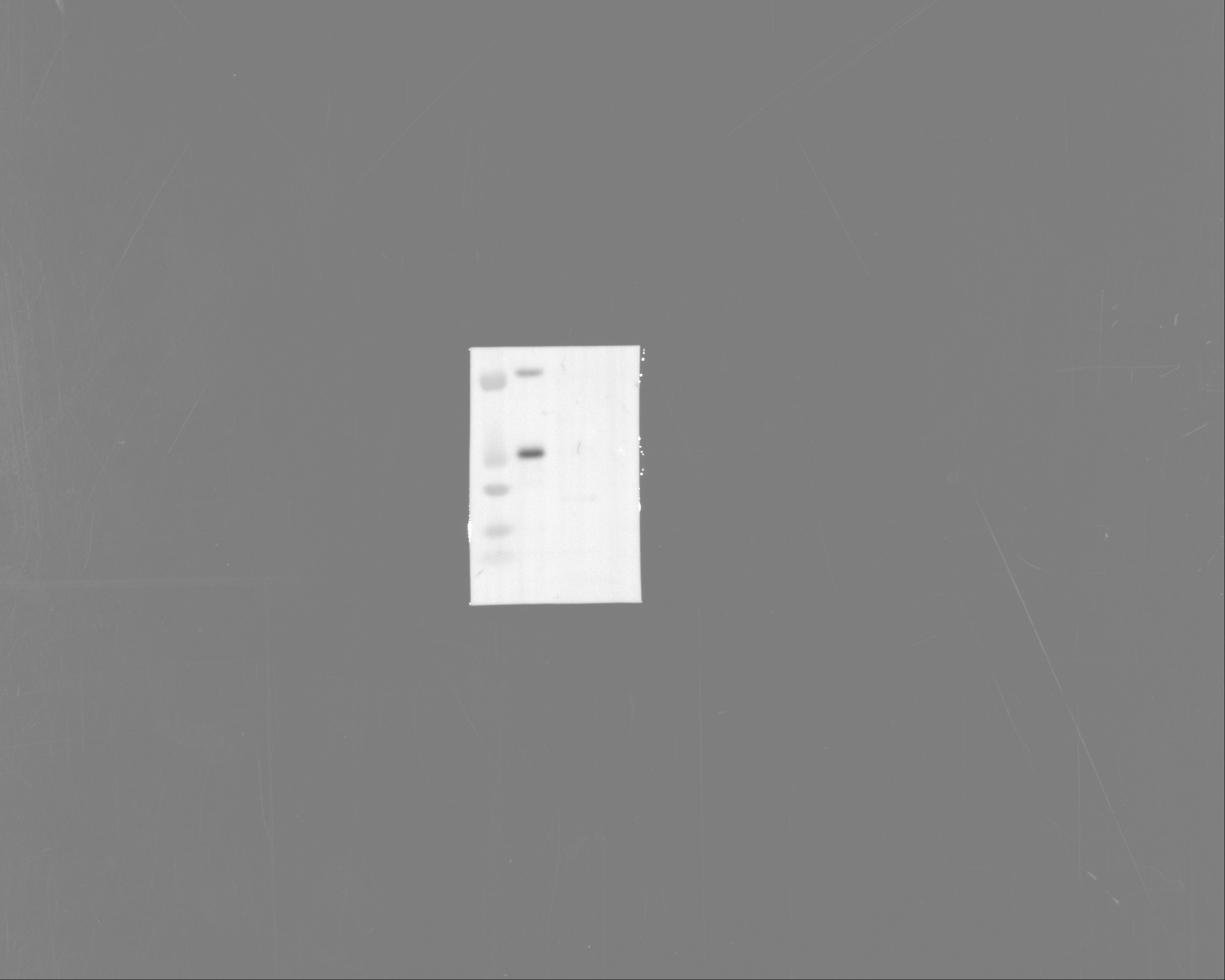

Supplement: Figure 3—figure supplement 1—source data 1. [file elife-86936-fig3-figsupp1-data1.zip › Figure 3 - Supplement 1 - Source Data 1/Uncropped Originals/Panel C - DnaJB6 blot.tif]

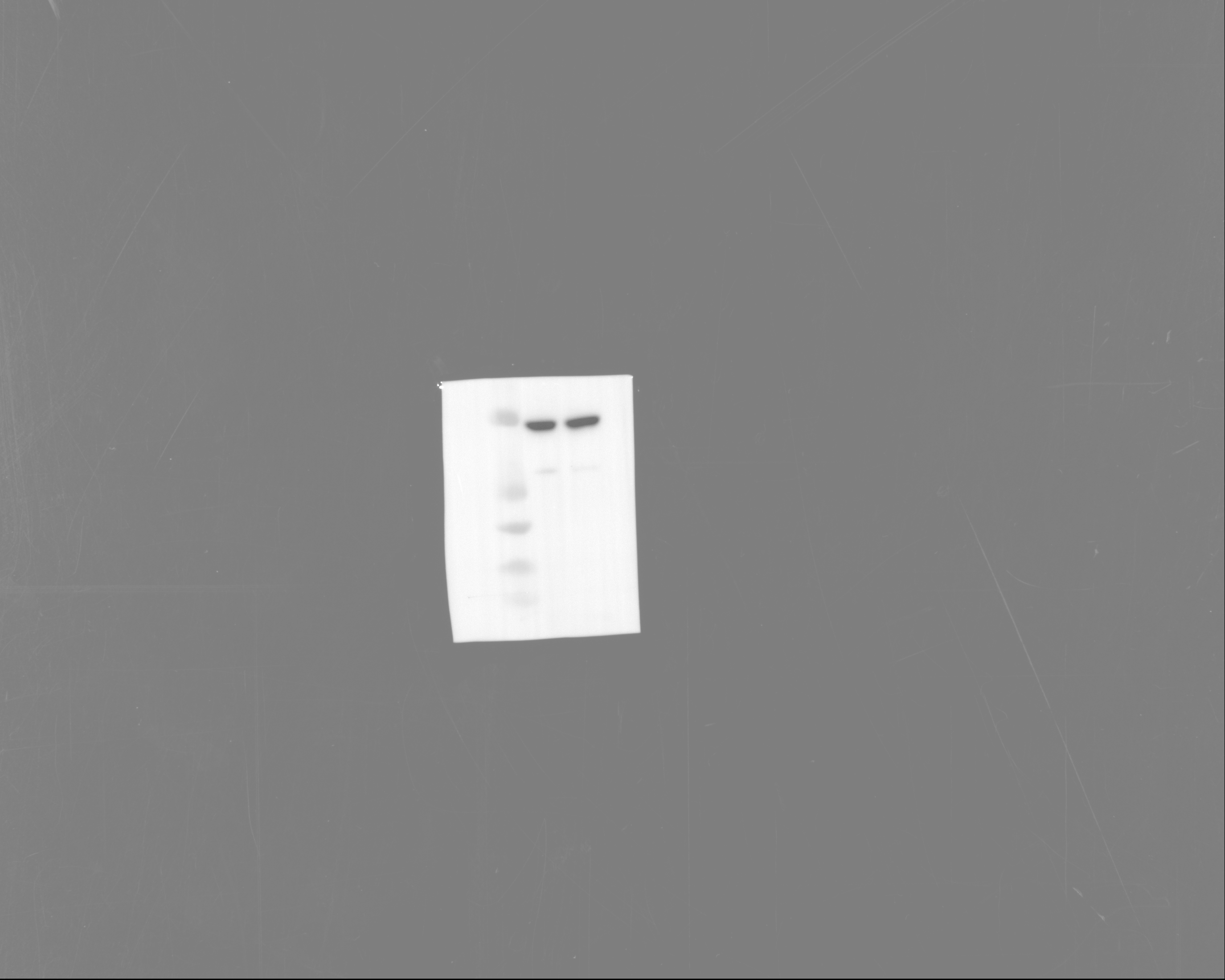

Supplement: Figure 3—figure supplement 1—source data 1. [file elife-86936-fig3-figsupp1-data1.zip › Figure 3 - Supplement 1 - Source Data 1/Uncropped Originals/Panel C - GAPDH blot.tif]

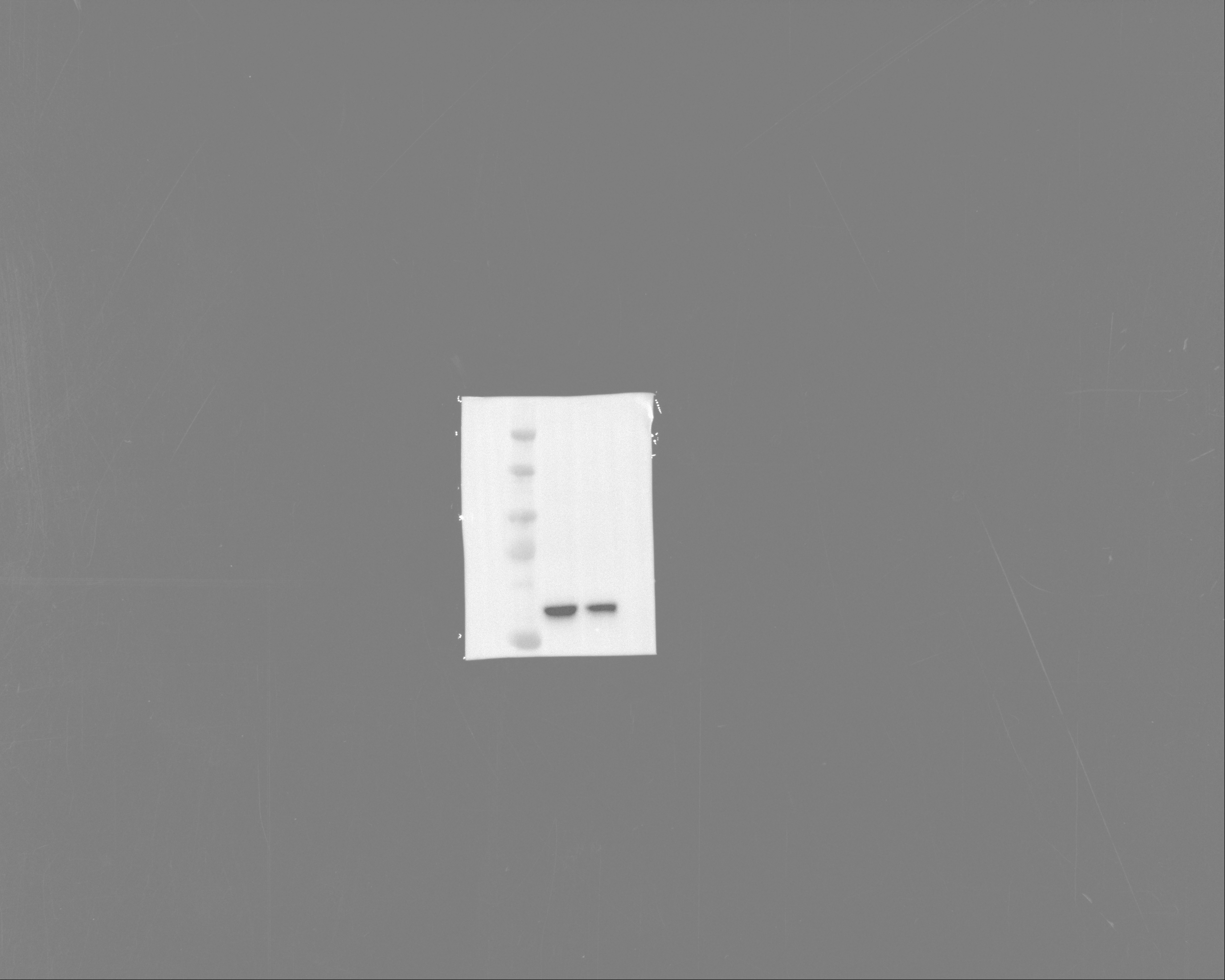

Supplement: Figure 3—figure supplement 1—source data 1. [file elife-86936-fig3-figsupp1-data1.zip › Figure 3 - Supplement 1 - Source Data 1/Uncropped Originals/Panel C - DnaJC7 blot.tif]

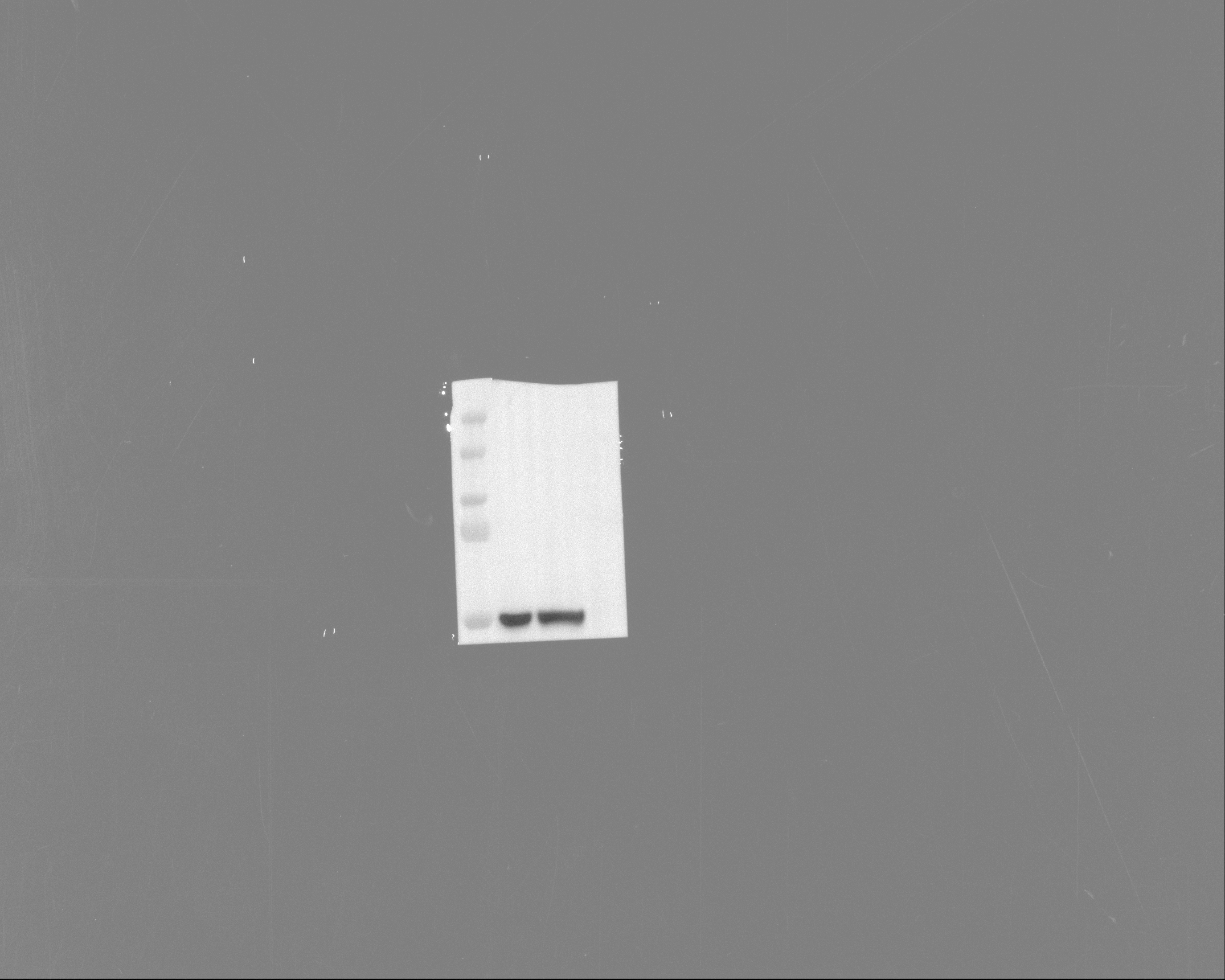

Supplement: Figure 3—figure supplement 1—source data 1. [file elife-86936-fig3-figsupp1-data1.zip › Figure 3 - Supplement 1 - Source Data 1/Uncropped Originals/Panel C - Tubulin blot.tif]

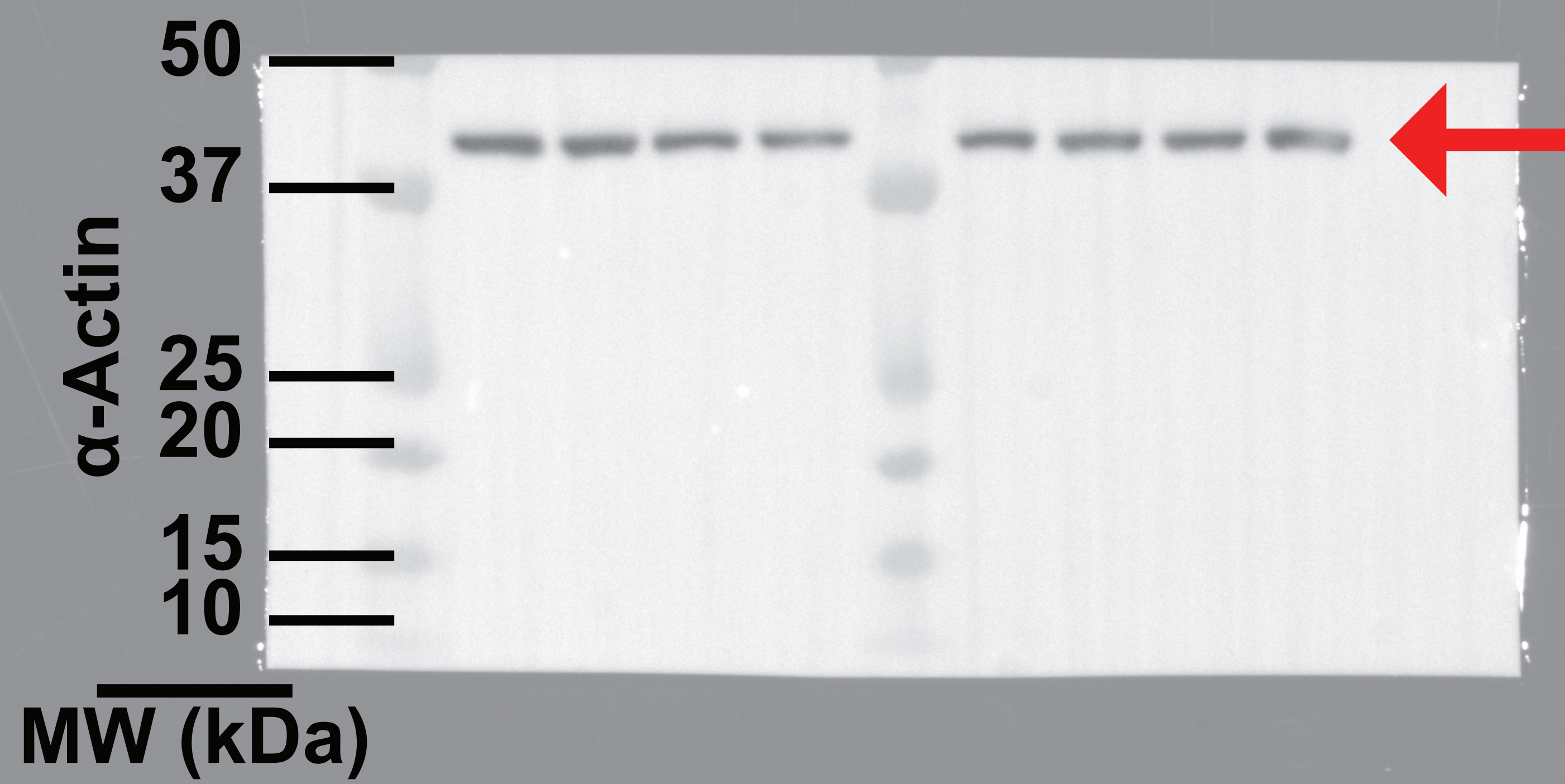

Supplement: Figure 5—figure supplement 1—source data 1. [file elife-86936-fig5-figsupp1-data1.zip › Figure 5 - Supplement 1 - Source Data 1/Uncropped Labelled/Panel B - actin blot.pdf]

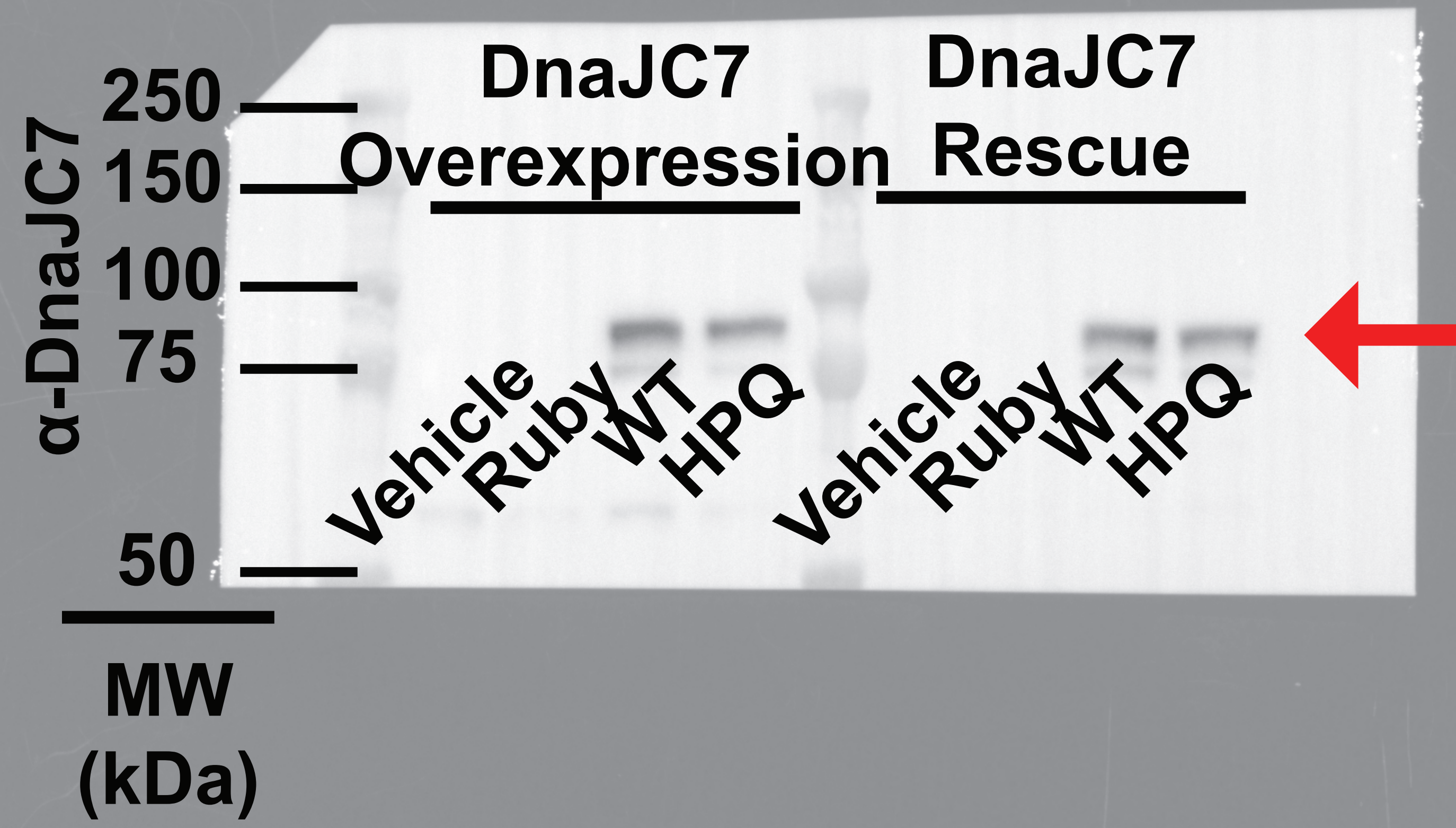

Supplement: Figure 5—figure supplement 1—source data 1. [file elife-86936-fig5-figsupp1-data1.zip › Figure 5 - Supplement 1 - Source Data 1/Uncropped Labelled/Panel B - DnaJC7 blot.pdf]

$\alpha$ -DnaJC7

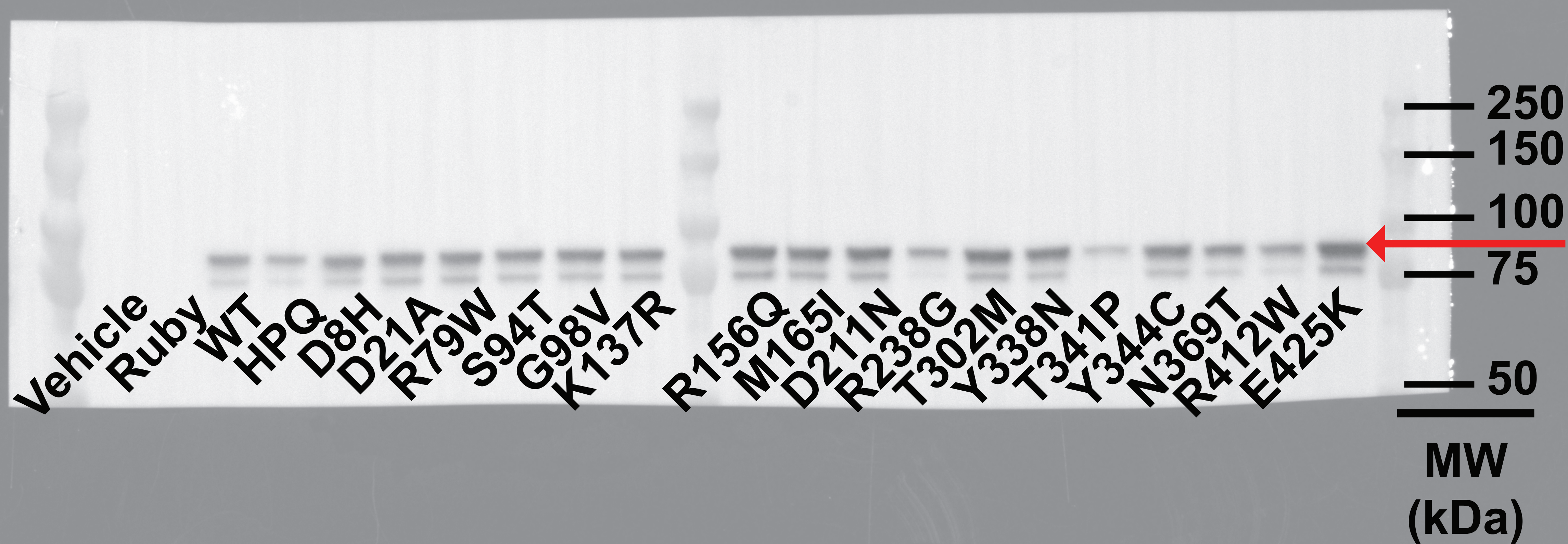

Supplement: Figure 5—figure supplement 1—source data 1. [file elife-86936-fig5-figsupp1-data1.zip › Figure 5 - Supplement 1 - Source Data 1/Uncropped Labelled/Panel C - DnaJC7 blot.pdf]

$\alpha$ -Actin

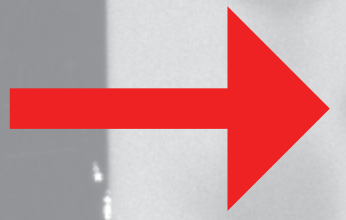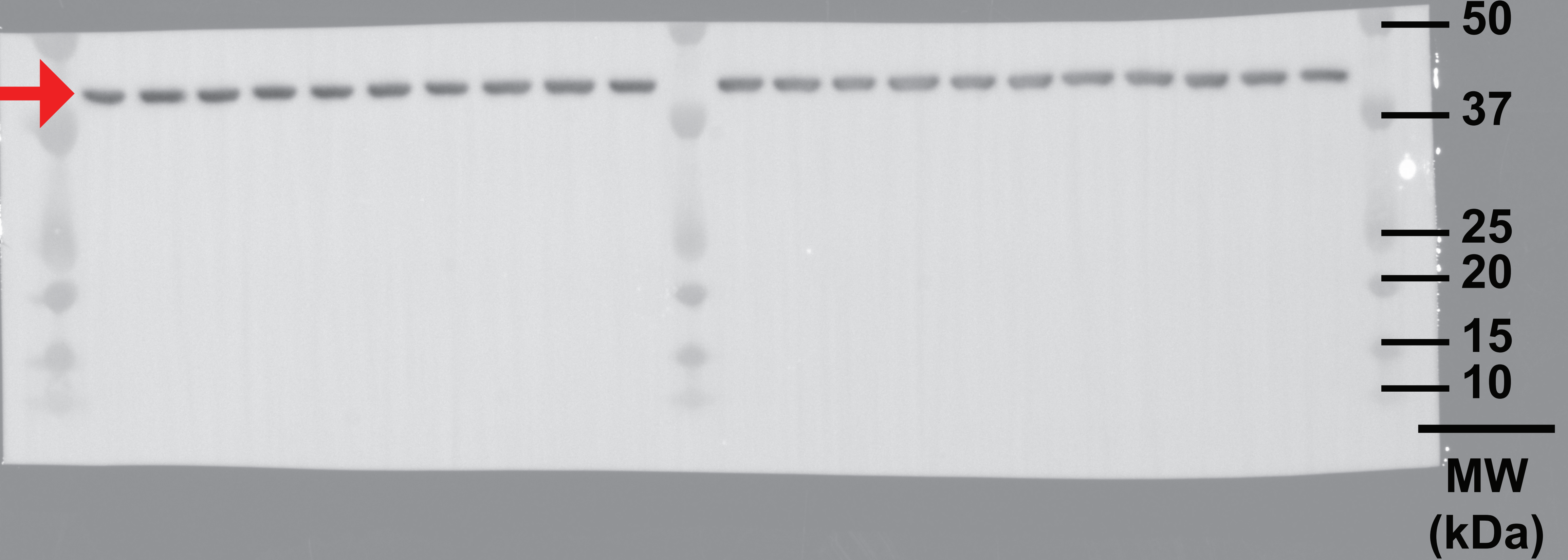

MW  
(kDa)

Supplement: Figure 5—figure supplement 1—source data 1. [file elife-86936-fig5-figsupp1-data1.zip › Figure 5 - Supplement 1 - Source Data 1/Uncropped Labelled/Panel C - actin blot.pdf]

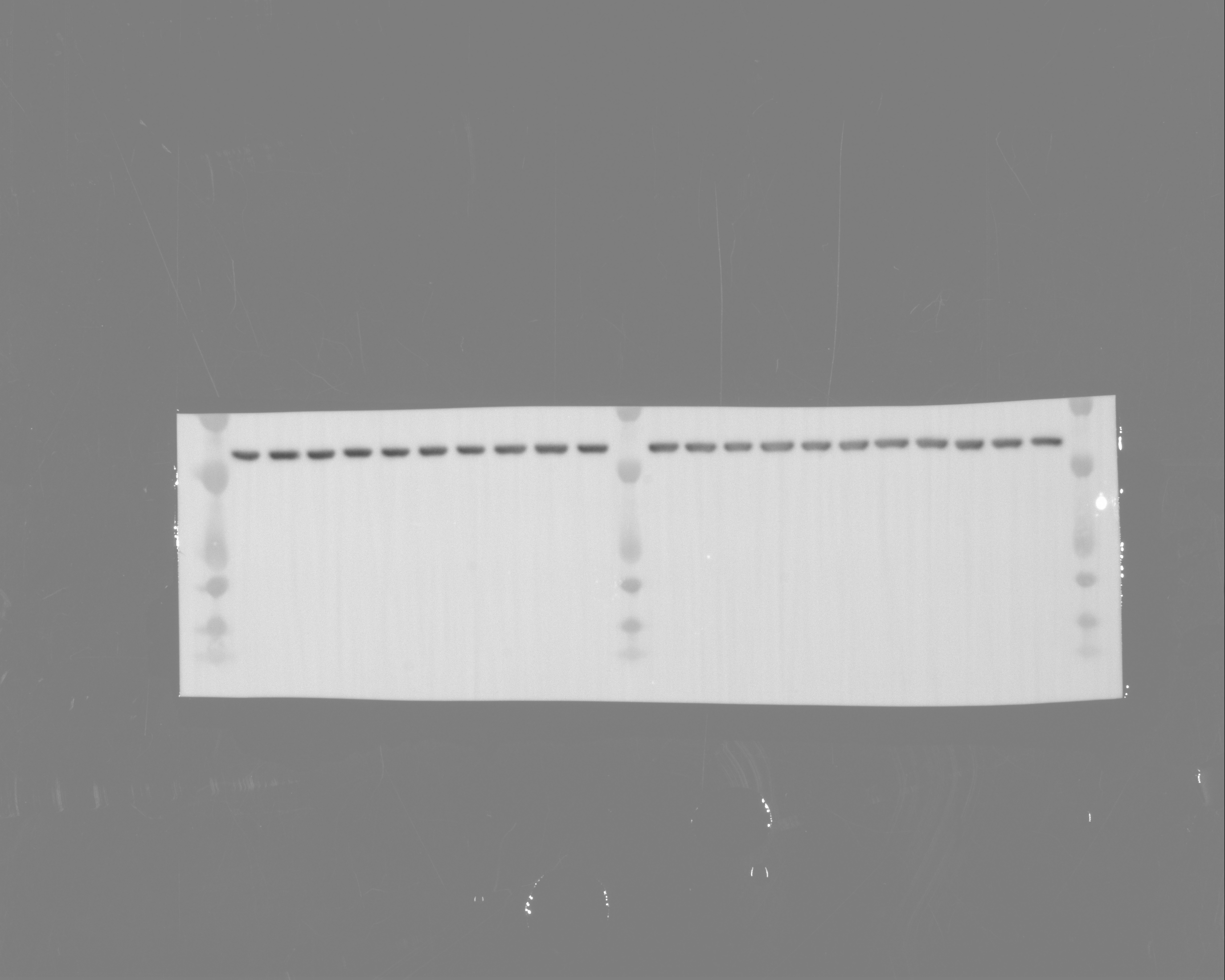

Supplement: Figure 5—figure supplement 1—source data 1. [file elife-86936-fig5-figsupp1-data1.zip › Figure 5 - Supplement 1 - Source Data 1/Uncropped Originals/Panel C - actin blot.tif]

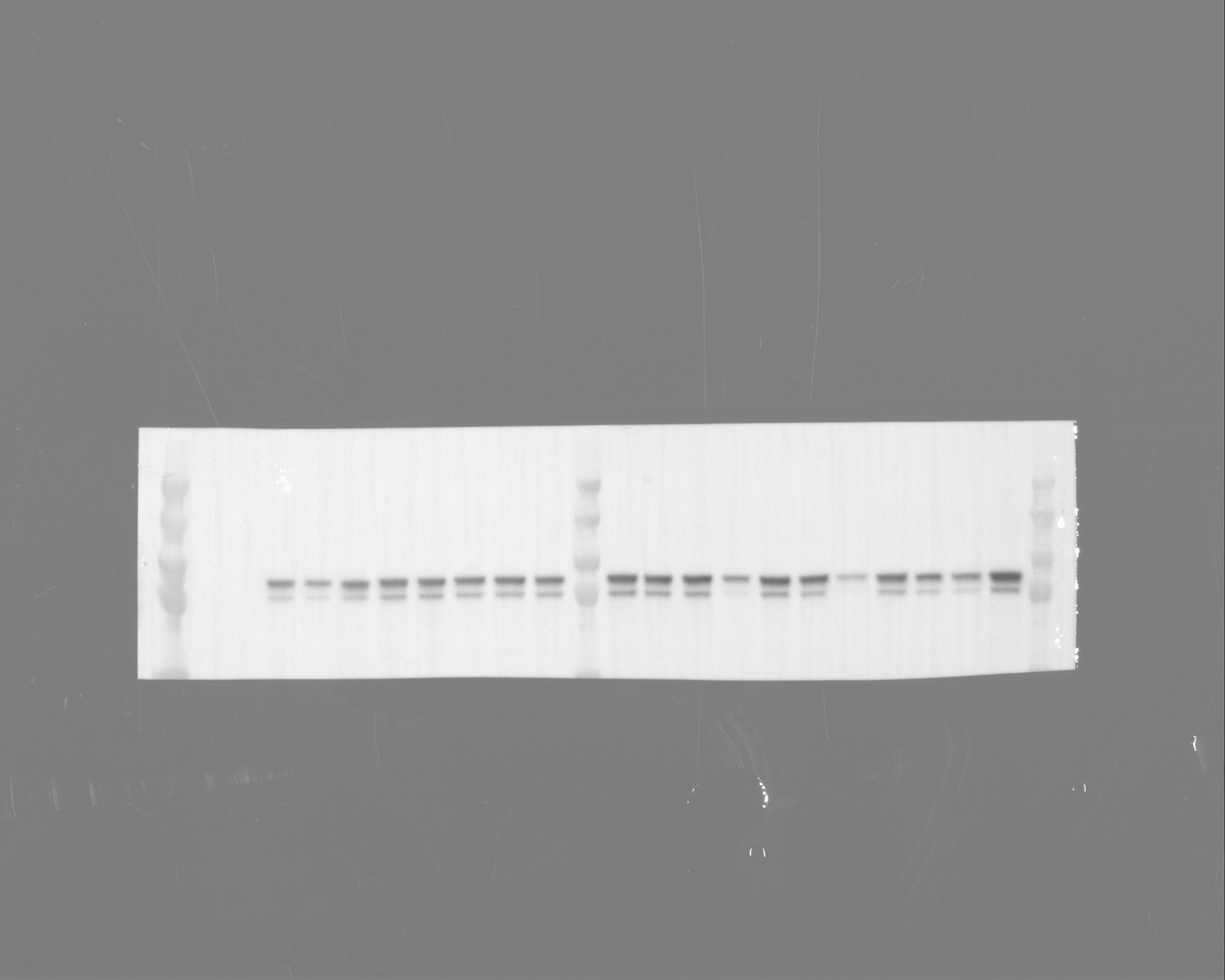

Supplement: Figure 5—figure supplement 1—source data 1. [file elife-86936-fig5-figsupp1-data1.zip › Figure 5 - Supplement 1 - Source Data 1/Uncropped Originals/Panel C - DnaJC7 blot.tif]

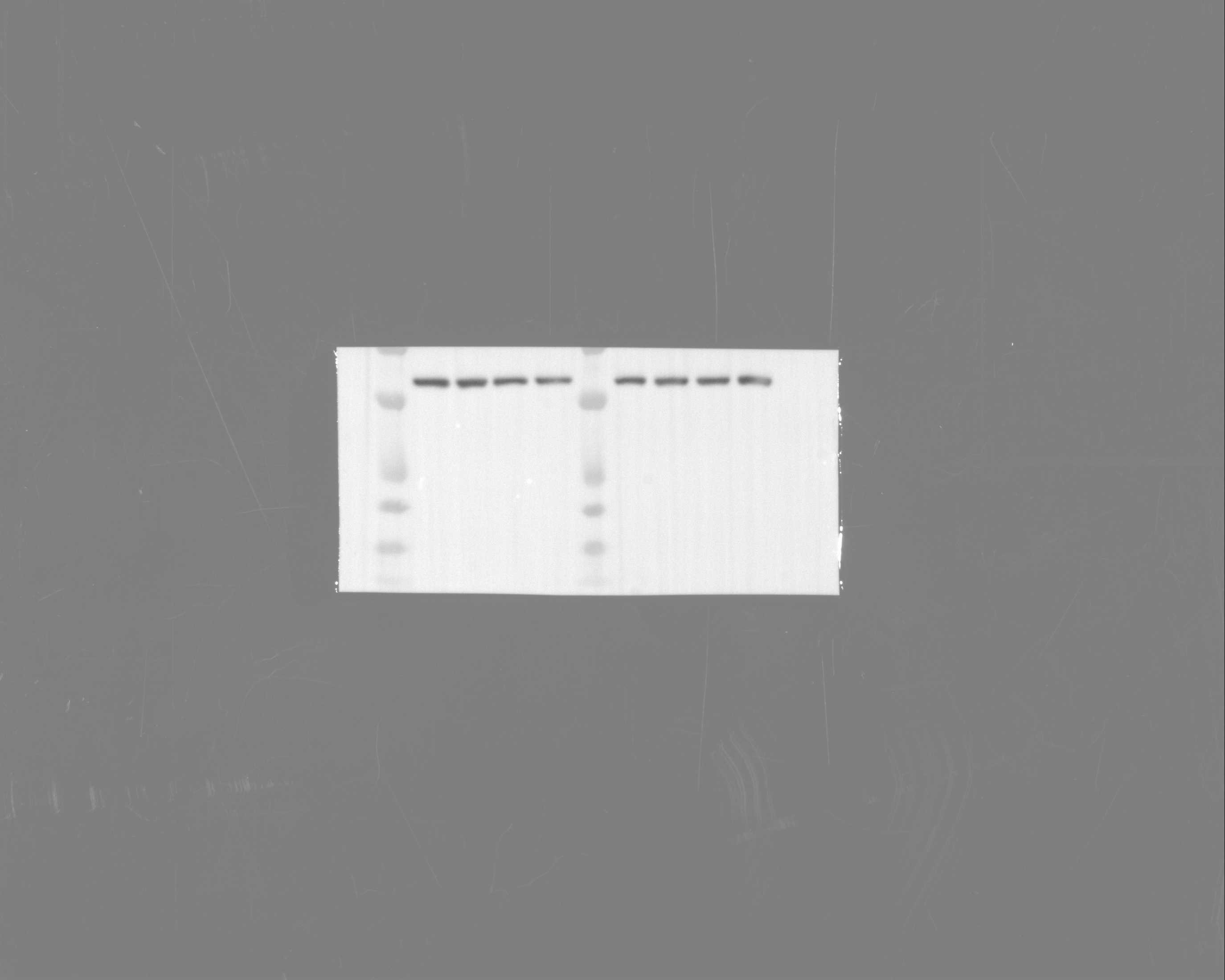

Supplement: Figure 5—figure supplement 1—source data 1. [file elife-86936-fig5-figsupp1-data1.zip › Figure 5 - Supplement 1 - Source Data 1/Uncropped Originals/Panel B - actin blot.tif]

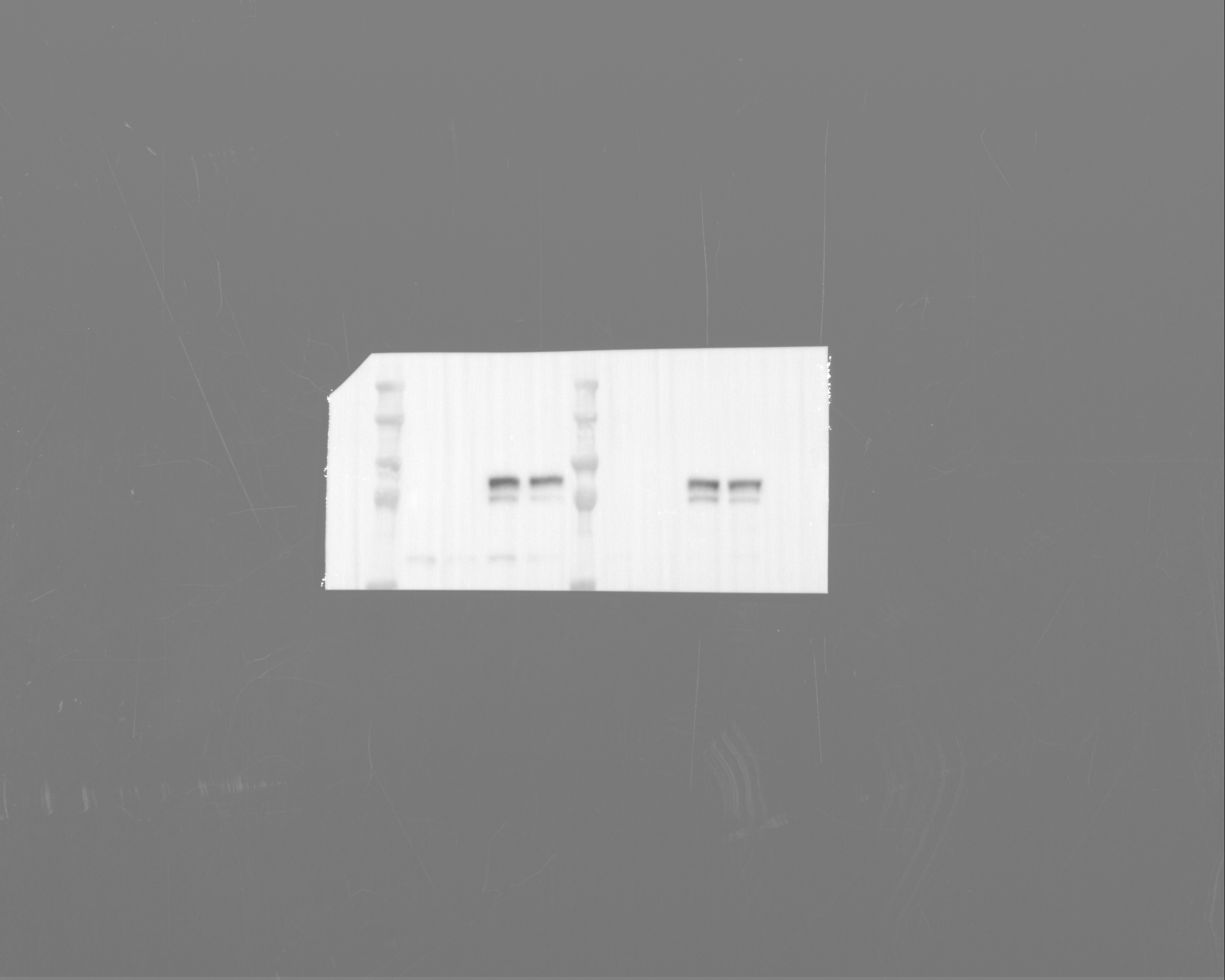

Supplement: Figure 5—figure supplement 1—source data 1. [file elife-86936-fig5-figsupp1-data1.zip › Figure 5 - Supplement 1 - Source Data 1/Uncropped Originals/Panel B - DnaJC7 blot.tif]
